# Role of HDL function and LDL atherogenicity on cardiovascular risk: A comprehensive examination

**DOI:** 10.1371/journal.pone.0218533

**Published:** 2019-06-27

**Authors:** Álvaro Hernáez, María Trinidad Soria-Florido, Helmut Schröder, Emilio Ros, Xavier Pintó, Ramón Estruch, Jordi Salas-Salvadó, Dolores Corella, Fernando Arós, Lluis Serra-Majem, Miguel Ángel Martínez-González, Miquel Fiol, José Lapetra, Roberto Elosua, Rosa María Lamuela-Raventós, Montserrat Fitó

**Affiliations:** 1 Cardiovascular Risk, Nutrition and Aging Research Unit, August Pi i Sunyer Biomedical Research Institute (IDIBAPS), Barcelona, Spain; 2 Blanquerna School of Life Sciences, Universitat Ramón Llull, Barcelona, Spain; 3 CIBER de Fisiopatología de la Obesidad y Nutrición (CIBEROBN), Instituto de Salud Carlos III (ISCIII), Madrid, Spain; 4 Cardiovascular Risk and Nutrition Research Group, Hospital del Mar Medical Research Institute (IMIM), Barcelona, Spain; 5 PhD Program in Food Sciences and Nutrition, Universitat de Barcelona, Barcelona, Spain; 6 CIBER de Epidemiología y Salud Pública (CIBERESP), ISCIII, Madrid, Spain; 7 Lipid Clinic, Endocrinology and Nutrition Service, Hospital Clínic, Barcelona, Spain; 8 Lipids and Vascular Risk Unit, Internal Medicine Service, Hospital Universitario de Bellvitge, Hospitalet de Llobregat, Spain; 9 Internal Medicine Service, Hospital Clínic, Barcelona, Spain; 10 Human Nutrition Unit, Hospital Universitari Sant Joan, Institut d’Investigació Sanitaria Pere Virgili, Universitat Rovira i Virgili, Reus, Spain; 11 Department of Preventive Medicine, Universidad de Valencia, Valencia, Spain; 12 Department of Cardiology, Hospital Universitario de Álava, Vitoria, Spain; 13 Department of Clinical Sciences & Research Institute of Biomedical and Health Sciences, Universidad de Las Palmas de Gran Canaria, Las Palmas, Spain; 14 Department of Preventive Medicine and Public Health, Universidad de Navarra, Pamplona, Spain; 15 Department of Nutrition, Harvard TH Chan School of Public Health, Boston, Massachusetts, United States of America; 16 Balearic Islands Health Research Institute, Hospital Son Espases, Palma de Mallorca, Spain; 17 Department of Family Medicine, Research Unit, Distrito Sanitario Atención Primaria Sevilla, Sevilla, Spain; 18 Cardiovascular Epidemiology and Genetics-REGICOR Research Group, Hospital del Mar Medical Research Institute (IMIM), Barcelona, Spain; 19 CIBER de Enfermedades Cardiovasculares (CIBERCV), ISCIII, Madrid, Spain; 20 Department of Nutrition and Bromatology, Faculty of Pharmacy, Universitat de Barcelona, Barcelona, Spain; Beijing Key Laboratory of Diabetes Prevention and Research, CHINA

## Abstract

**Background:**

High-density lipoprotein (HDL) functionality and low-density lipoprotein (LDL) atherogenic traits can describe the role of both particles on cardiovascular diseases more accurately than HDL- or LDL-cholesterol levels. However, it is unclear how these lipoprotein properties are particularly affected by different cardiovascular risk factors.

**Objective:**

To determine which lipoprotein properties are associated with greater cardiovascular risk scores and each cardiovascular risk factor.

**Methods:**

In two cross-sectional baseline samples of PREDIMED trial volunteers, we assessed the associations of HDL functionality (*N* = 296) and LDL atherogenicity traits (*N* = 210) with: 1) the 10-year predicted coronary risk (according to the Framingham-REGICOR score), and 2) classical cardiovascular risk factors.

**Results:**

Greater cardiovascular risk scores were associated with low cholesterol efflux values; oxidized, triglyceride-rich, small HDL particles; and small LDLs with low resistance against oxidation (*P*-trend<0.05, all). After adjusting for the rest of risk factors; 1) type-2 diabetic individuals presented smaller and more oxidized LDLs (*P*<0.026, all); 2) dyslipidemic participants had smaller HDLs with an impaired capacity to metabolize cholesterol (*P*<0.035, all); 3) high body mass index values were associated to lower HDL and LDL size and a lower HDL capacity to esterify cholesterol (*P*<0.037, all); 4) men presented a greater HDL oxidation and lower HDL vasodilatory capacity (*P*<0.046, all); and 5) greater ages were related to small, oxidized, cytotoxic LDL particles (*P*<0.037, all).

**Conclusions:**

Dysfunctional HDL and atherogenic LDL particles are present in high cardiovascular risk patients. Dyslipidemia and male sex are predominantly linked to HDL dysfunctionality, whilst diabetes and advanced age are associated with LDL atherogenicity.

## Introduction

Low levels of high-density lipoprotein (HDL) cholesterol (HDL-C) and high concentrations of low-density lipoprotein (LDL) cholesterol (LDL-C) are traditionally related to a greater risk of suffering a cardiovascular event [1]. However, HDL functions could reflect the protective role of the lipoprotein better than HDL-C levels [2], and LDL characteristics provide further information on the residual atherogenic risk of these particles beyond mere LDL-C concentrations [3,4].

Both lipoprotein traits have been shown to be associated with high cardiovascular risk states in very diverse ways. On the one hand, regarding HDL functions: 1) cholesterol efflux capacity (HDL capacity to pick up cholesterol from peripheral cells) has demonstrated to be inversely related with the incidence of cardiovascular events (and shown to predict these outcomes more accurately than HDL-C concentrations) [5]; 2) deficiencies in the biological function of two enzymes related to the metabolism of cholesterol in HDLs, lecithin-cholesterol acyltransferase (LCAT, responsible for the esterification and internalization of free cholesterol after cholesterol efflux) and cholesteryl ester transfer protein (CETP, responsible for the exchange of cholesterol from HDLs to other lipoproteins), have shown to be linked to modest increments (LCAT) or decrements (CETP) in the incidence of cardiovascular events [6,7], although the effect of modifying these activities in other studies has not been shown to be conclusive [8]; 3) the activity of paraoxonase-1 (PON1, an essential antioxidant HDL enzyme) has been inversely associated with cardiovascular diseases incidence in some works [9] but not in others [10]; 4) HDLs are also thought to promote endothelial protection and are linked to a greater release of nitric oxide from endothelial cells [11], being this property transiently impaired in acute coronary events [12]; finally, 5) HDL oxidation and its global lipid composition, although being related to several aspects of a dysfunctional lipoprotein profile (a decreased capacity to perform HDL biological functions or a decreased HDL stability) [13–16], have not been associated with high cardiovascular risk (CVR) states as clearly as other HDL functional traits. On the other hand, regarding LDL atherogenic characteristics: 1) circulating levels of oxidized LDLs are directly related with incidence of coronary diseases and all-cause mortality [4,17], whilst low LDL resistance against oxidative modifications of the particle has been linked with subclinical atherosclerosis and is present in high CVR subjects [18,19]; 2) small LDL particles (a characteristic deeply interrelated with a pro-atherogenic LDL profile, which can be indirectly measured by the ratio between LDL-C and apolipoprotein B–ApoB–levels in circulation [20]), have been associated with a greater incidence of cardiovascular events [21]; and 3) compositional changes of LDL particles such as increases in their remnant triglyceride content tend to increase ApoB-100 instability on LDL surface (which may lead to an inefficient binding to LDL receptors) and have shown to be increased in coronary artery disease patients [22,23]. The aim of this study was to determine the independent associations of HDL functionality and LDL atherogenic characteristics with: 1) the 10-year predicted risk of suffering a coronary event (the Framingham-REGICOR CVR score), and 2) the most prevalent CVRFs (diabetes, dyslipidemia, excess body weight, hypertension, and smoking habit), age, and sex, in high CVR individuals.

## Materials and methods

### Study population

This study was a cross-sectional analysis in two sub-samples of volunteers from the PREDIMED Study [24,25] at the baseline visit: one sample for the evaluation of HDL-related variables (*N* = 296) [26] and another for the assessment of LDL atherogenic traits (*N* = 210) [27]. The sample for the study of HDL-related parameters included the one in which the LDL-related characteristics were assessed. In these populations, we registered the values of: 1) general clinical variables (age, sex, body weight, height, blood pressure, and biochemical profile); 2) drug use; 3) adherence to a Mediterranean Diet, by means of the Mediterranean diet Score; 4) levels of physical activity according to the Minnesota Leisure Time physical Activity questionnaire; and 5) smoking habit [24,28]. In individuals aged 35–74, we calculated 10-year predicted risk of developing a future coronary event as the CVR scores according to the Framingham-REGICOR equation validated for the Spanish population (considering age and sex, presence of diabetes and tobacco habit, total and HDL-C levels, and blood pressure) [29]. Type-II diabetes mellitus was defined as the presence of an abnormal glucose metabolism or use of anti-diabetic drugs. Dyslipidemia was defined as the presence of total cholesterol levels ≥200 mg/dL or use of statins and triglyceride levels ≥150mg/dL. Hypertension was defined as the presence of systolic blood pressure levels ≥140 mmHg, diastolic blood pressure levels ≥90 mmHg, or use of anti-hypertensive drugs. Body mass index (BMI) was calculated as the ratio between weight (kg) and height squared (m^2^) [24].

Volunteers provided written informed consent before entering the trial. The study protocol conforms to the ethical guidelines of the 1975 Declaration of Helsinki, was approved by the local Research and Ethics Committee, and was registered with the International Standard Randomized Controlled Trial Number ISRCTN35739639. Its details have been previously published [24, 25].

### HDL functionality determinations

We first isolated HDL particles from plasma by density gradient ultracentrifugation (isolated HDL fraction) [26,30] and polyethylene glycol-induced precipitation of apolipoprotein B (ApoB)-containing lipoproteins (ApoB-depleted plasma samples) [26]. Plasma, serum, isolated HDL, and ApoB-depleted plasma samples were stored at -80°C until use. We analyzed the participants’ lipid profile (triglycerides, cholesterol, HDL-C, and apolipoprotein A-I–ApoA-I–) in an ABX-Pentra 400 autoanalyzer (Horiba ABX) [26]. We determined cholesterol efflux capacity (HDL ability to pick up the cholesterol excess from cells) in a model of human THP-1 monocyte-derived macrophages treated with ApoB-depleted plasma samples [26]. We computed the ability of HDL lipoproteins to esterify cholesterol as the percentage of esterified cholesterol in isolated HDL particles/lecithin cholesterol acyltransferase quantity in plasma [26]. We determined the function of cholesteryl ester transfer protein (CETP) in plasma [26,30] and the arylesterase activity of paraoxonase-1 (PON1) in serum [26] by commercial kits. We assessed HDL vasodilatory capacity as the HDL-induced increment in the production of nitric oxide in a human umbilical vein endothelial cell model treated with ApoB-depleted plasma samples [26]. We determined the oxidation of HDL particles as the equivalents of malondialdehyde per mg/dL of cholesterol in ApoB-depleted plasma samples [26]. We examined the lipid composition of the isolated HDL fraction in an ABX-Pentra 400 autoanalyzer (Horiba ABX) and, from these data, we calculated the triglyceride/esterified cholesterol ratio in HDL particles (“triglycerides in HDL core”) [26,30]. Finally, we assessed HDL size distribution by LipoPrint technology (Quantimetrix) in plasma [26,30]. With the percentages of large and small HDL particles (HDL2 and HDL3, respectively), we calculated the HDL2/HDL3 ratio.

### LDL atherogenic traits

We first isolated LDL lipoproteins from plasma samples by density gradient ultracentrifugation [27,31] and stored them at -80°C until use. From the values of the participants’ lipid profile, we calculated LDL-C levels according to the Friedewald formula (whenever triglycerides were <300 mg/dL) [27,31]. We quantified ApoB in an ABX-Pentra 400 autoanalyzer (Horiba ABX) in plasma [27,31]B. We measured LDL resistance against oxidation (LDL lag time) from the kinetics of formation of conjugated dienes (oxidized lipid forms) in isolated LDL samples in a pro-oxidant environment [27,31]. We assessed the oxidation of LDL lipoproteins as the equivalents of malondialdehyde per mg/dL of cholesterol in isolated LDL samples [27]. From the lipid profile values, we calculated an approximation to LDL average size (the LDL-C/ApoB ratio) [27]. We determined the lipid composition of isolated LDL particles in an ABX-Pentra 400 autoanalyzer (Horiba ABX) and, from these data, we calculated the triglyceride/total cholesterol ratio in isolated LDL samples. Finally, we assessed LDL *ex vivo* cytotoxicity in a THP-1 monocyte-derived macrophage model as previously described [27].

### Sample size

Accepting a type I error of 0.05, a type II error of 0.2, and a 1% loss rate in a two-sided contrast, sample sizes of 196 and 140 participants provide sufficient statistical power to determine that Pearson’s correlation coefficients ≥0.2 and ≥0.237 (for HDL- and LDL-related variables, respectively) were significantly different from zero. Sample sizes were increased by 50%, up to 294 and 210 subjects, to allow adjustments for different covariates.

### Statistical analyses

We first assessed the distribution of continuous variables using normality plots and histograms.

To study the association between lipoprotein traits and CVR, we first compared the means of HDL- and LDL-related variables among the CVR score groups (low risk–CVR score <5–, moderate risk–CVR score ≥5 and <10–, and high risk–CVR score ≥10–) using a one-way ANOVA for normally-distributed variables and a Kruskal-Wallis test for non-normally distributed ones. To determine possible linear associations between the CVR score group and the means or medians of lipoprotein-related variables, we performed Pearson’s or Spearman’s tests, respectively, to calculate *P*-trend values.

We assessed the differences in the values of lipoprotein characteristics due to classical CVRFs (presence of diabetes, dyslipidemia, hypertension, and tobacco use–categorical variables–; and greater values of BMI–continuous variable–), sex, and age (continuous variable), in three multivariate linear regression models. Model 1 was non-adjusted. To determine the independent effect of each of these traits on lipoprotein characteristics, model 2 was adjusted for the rest of the previous factors, study site, adherence to the Mediterranean diet, and levels of physical activity. Finally, model 3 included HDL-C or LDL-C levels as an extra co-variate, in order to exclude the effect of lipoprotein cholesterol from the previous associations.

We accepted any two-sided *P*-value <0.05 as significant. We executed the previously described analyses in R Software, version 3.4.1 (*R*: *A language and environment for statistical computing*. *R Foundation for Statistical Computing*. *Vienna*, *Austria*).

## Results

### Participants

In accordance to the high CVR profile of the volunteers in the HDL functionality and LDL atherogenicity subsamples, subjects were 65.9 and 65.4 years old, 49.0 and 51.4% of the participants were male, 49.0 and 47.1% were diabetic, 33.4 and 33.3% were under glucose-lowering treatment, 77.4 and 79.0% were dyslipidemic, 44.9 and 43.3% were statin users, 78.7 and 82.4% were hypertensive, 65.9 and 70.0% were under antihypertensive treatment, 44.9 and 44.8% were obese, and 12.5 and 13.8% were smokers, respectively (Table 1).

**Table 1:** Baseline characteristics of the HDL functionality and LDL atherogenicity subsamples from volunteers of the PREDIMED study.

	HDL functionality subsample (*n* = 296)^a^	LDL atherogenicity subsample (*n* = 210)^a^
Age (years)	65.9 (6.43)	65.4 (6.60)
Male sex (%)	49.0%	51.4%
Diabetic individuals (%)	49.0%	47.1%
Glucose-lowering therapy users (%)	33.4%	33.3%
Dyslipidemic individuals (%)	77.4%	79.0%
Statin users (%)	44.9%	43.3%
Hypertensive individuals (%)	78.7%	82.4%
Antihypertensive therapy users (%)	65.9%	70.0%
Obesity (%)	44.9%	44.8%
Smoking habit:		
Never smokers (%)	58.1%	56.2%
Smokers (%)	12.5%	13.8%
Former smokers (%)	29.4%	30.0%

^a^HDL indicates high-density lipoprotein; LDL, low-density lipoprotein

### HDL functionality, LDL atherogenicity, and CVR categories

**Fig 1:**
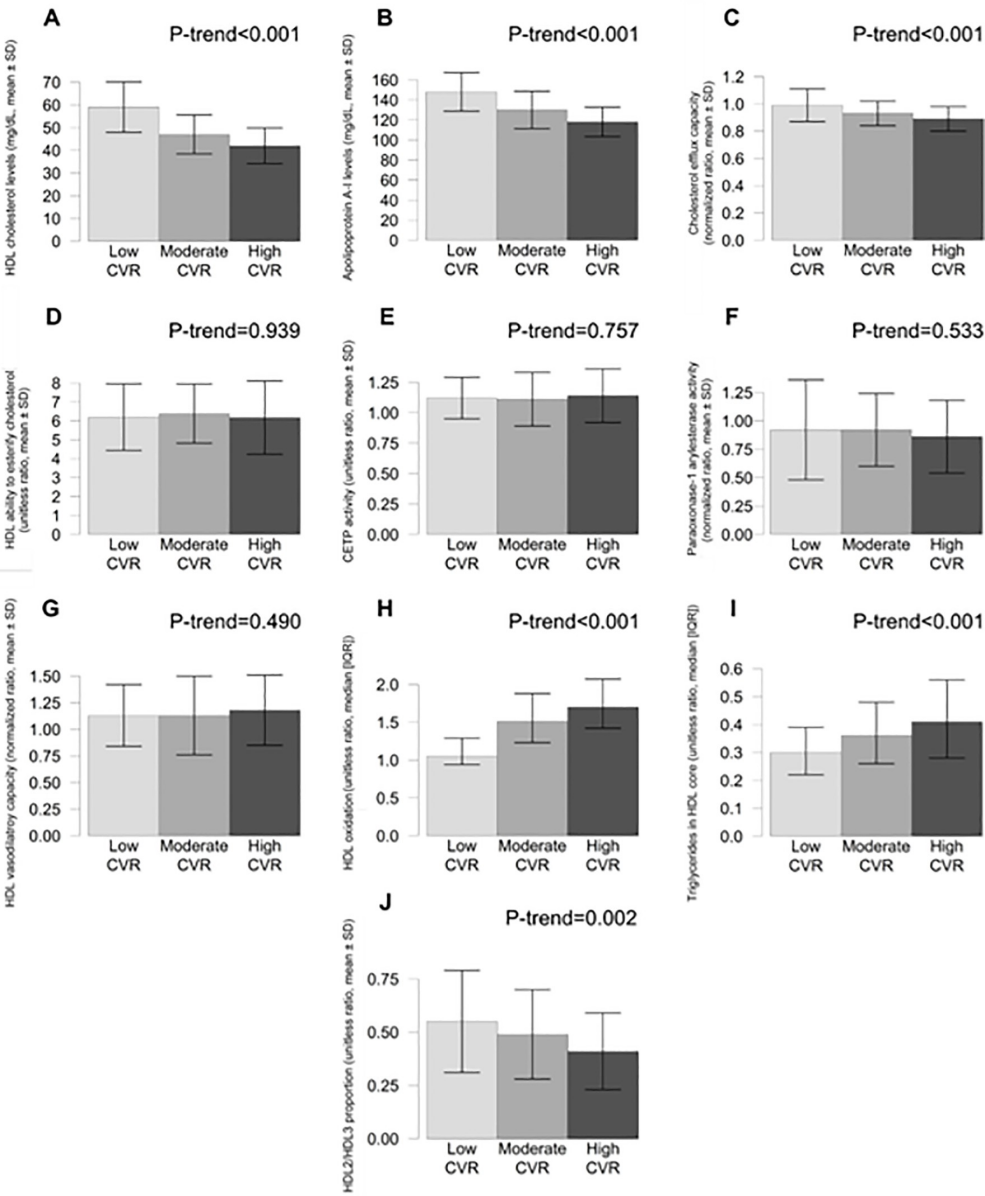
Values of HDL functionality variables in the three groups according to CVR scores. Low CVR (score<5, *N* = 77), moderate CVR (score ≥5 and <10, *N* = 115), and high CVR (score ≥10, *N* = 52). CVR indicates cardiovascular risk; HDL, high-density lipoprotein.

Regarding HDL-related traits, high CVR was associated with low HDL-C and ApoA-I levels, low cholesterol efflux values, high HDL oxidation, high content of triglycerides in HDL core (*P*<0.001 in the five previous cases), and low values of the HDL2/HDL3 ratio (smaller HDL size) (*P* = 0.002) (Fig 1).

**Fig 2:**
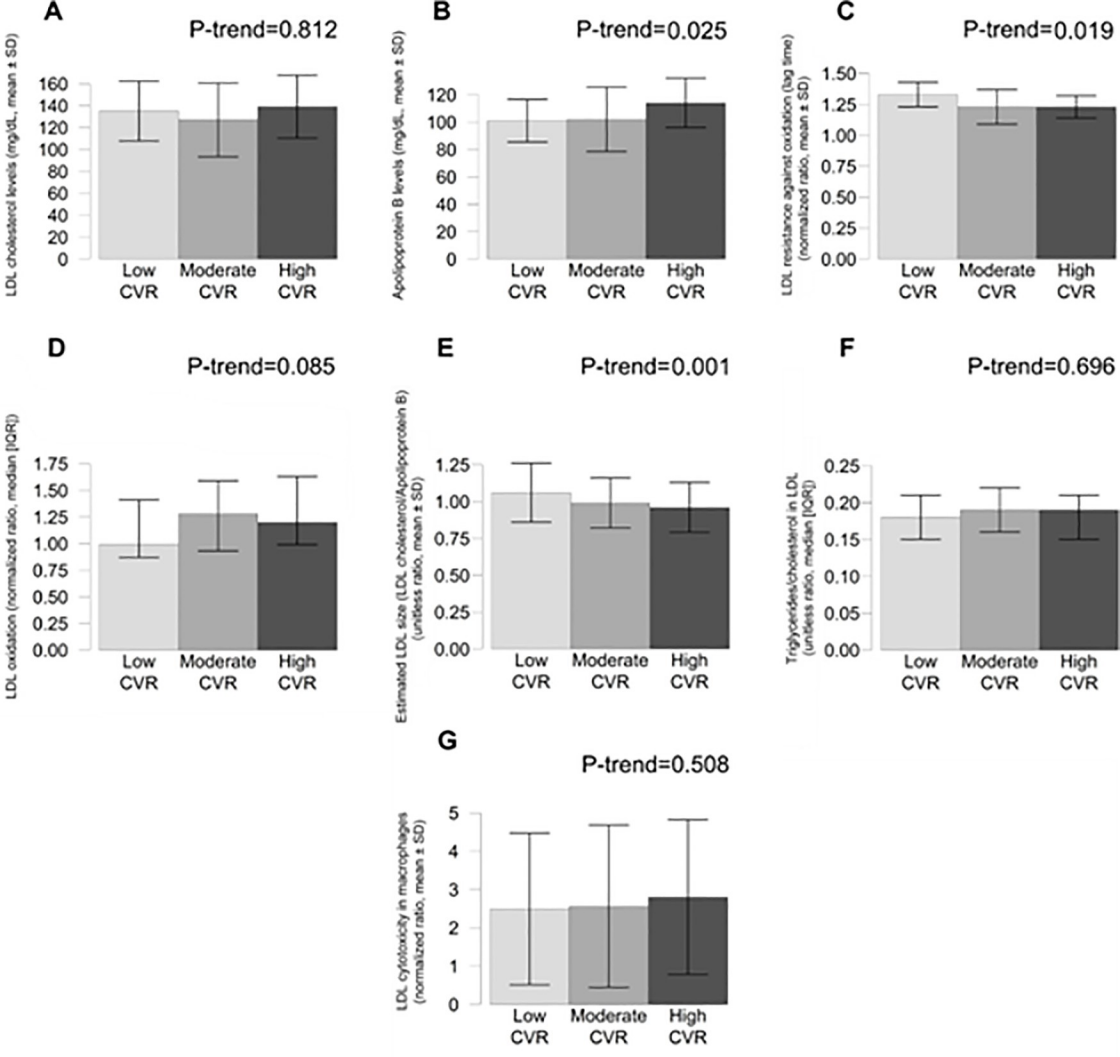
Values of LDL functionality variables in the three groups according to CVR scores. Low CVR (score<5, *N* = 48), moderate CVR (score ≥5 and <10, *N* = 88), and high CVR (score ≥10, *N* = 38). CVR indicates cardiovascular risk; LDL, low-density lipoprotein. Error bars depict standard deviations.

Regarding LDL-related variables, high CVR was associated with high ApoB levels (*P* = 0.025) (but not with significant differences in LDL-C levels, *P*>0.05), low LDL resistance against oxidation (low LDL lag time values) (*P* = 0.019), and low estimated LDL size (*P* = 0.001), and with a borderline significant trend towards high LDL oxidation (*P* = 0.085) (**Fig 2**). Error bars depict standard deviations.

### Individual effects of CVRFs on lipoprotein traits in high CVR subjects

**Fig 3:**
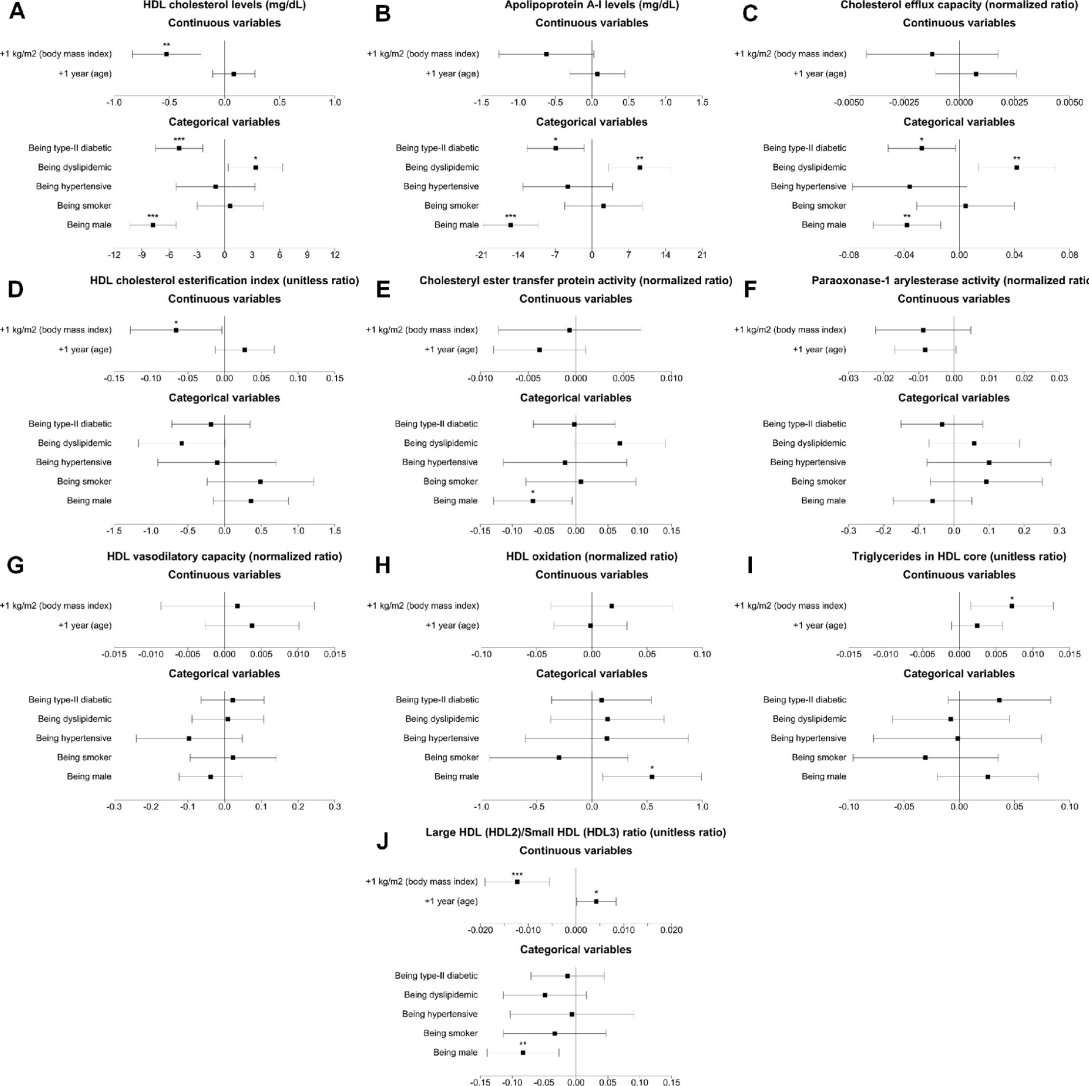
Forest plots of the associations of cardiovascular risk factors with HDL functional properties. HDL indicates high-density lipoprotein. *: *P*<0.05; **: *P*<0.01; ***: *P*<0.001.

**Fig 4:**
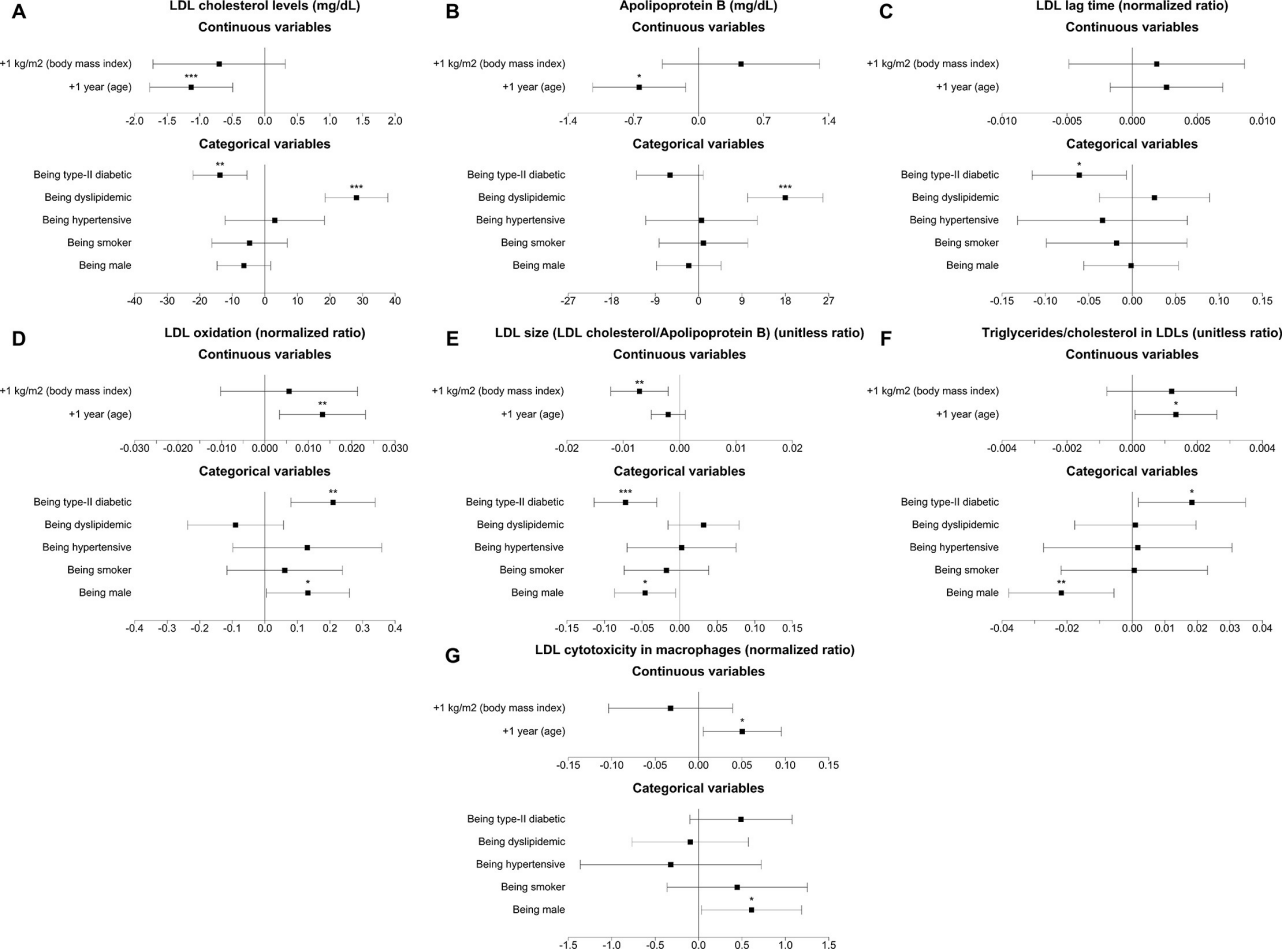
Forest plots of the associations of cardiovascular risk factors with LDL pro-atherogenic traits. LDL indicates low-density lipoprotein. *: *P*<0.05; **: *P*<0.01; ***: *P*<0.001.

As observed in Fig 3, presence of type-II diabetes was associated with low HDL-C (*P*<0.001) and ApoA-I levels (*P* = 0.004) and low cholesterol efflux capacity values (*P* = 0.001). Regarding LDL properties (see Fig 4), diabetes was related to low LDL-C (*P*<0.001) levels, greater LDL oxidation (*P*<0.001) and lower LDL resistance against oxidation (*P* = 0.007), lower estimated LDL size (*P*<0.001) and a greater LDL triglyceride content (*P* = 0.032).

Dyslipidemia was related to greater cholesterol and apolipoprotein levels (*P*<0.05, all) and high cholesterol efflux capacity values (*P*<0.001) (Figs 3 and 4).

High BMI values were associated with low HDL-C levels (*P* = 0.006), low HDL capacity to esterify cholesterol (*P* = 0.008), smaller HDL size (*P*<0.001), and a greater triglyceride content in the HDL core (*P* = 0.014). High BMI values were also linked to a lower estimated LDL size after adjusting for all CVRFs. (*P* = 0.004).

Hypertension was not particularly associated with an abnormal lipoprotein profile (data not shown), although it was independently linked to a borderline significant trend towards low cholesterol efflux capacity values when adjusted for all classical CVRFs (*P* = 0.090). Finally, being smoker was also unrelated to differences in any lipoprotein characteristic (data not shown).

The exact coefficients of the associations of cardiovascular risk factors with lipoprotein properties for the three models performed are available in S1 Table.

### Effects of sex and age on lipoprotein traits in high CVR patients

Male sex was associated with a highly dysfunctional HDL profile (Fig 2 and S2 Table): after adjusting for all classical CVRFs, men had lower HDL-C and ApoA-I levels (*P*<0.001, both), lower cholesterol efflux capacity values (*P* = 0.003), lower CETP activities (*P* = 0.033), greater HDL oxidation (*P* = 0.018), and smaller HDL particles (*P* = 0.004). Regarding LDLs, after adjusting for CVRFs, male sex was linked to lower estimated LDL size (*P* = 0.028), and to greater LDL oxidation (*P* = 0.044), cholesterol content (*P* = 0.009), and cytotoxicity (*P* = 0.040). Finally, regarding HDL functionality, greater ages were related to larger HDL particles (*P*<0.001). Despite greater age being linked to low LDL-C and ApoB levels (*P*<0.001), it was also independently associated with a greater LDL oxidation (*P* = 0.009), a greater LDL triglyceride content (*P* = 0.037), and greater LDL cytotoxic potential (*P* = 0.030) when adjusting for all classical CVRFs.

## Discussion

Our data show that dysfunctional HDL and atherogenic LDL particles are associated with greater CVR scores and particularly impaired in certain high CVR subjects (diabetic, dyslipidemic, with excess weight, male, and older) in the first systematic, comprehensive association analysis performed to date.

HDL functions are intimately related to CVR according to previous human studies. In our dataset, we have observed an association between high CVR and low cholesterol efflux, high HDL oxidation, high triglyceride content in HDL core, and smaller HDL size. Cholesterol efflux capacity has already shown to be related to high subclinical atherosclerosis and incidence of cardiovascular diseases [5,32]. Regarding HDL oxidation, it has been previously associated with high CVR states [13] as well as with decreased cholesterol efflux capacity values [14]. A high triglyceride content in the HDL core has been shown to contribute to HDL instability; It leads to an imbalance in the electrostatic relationships of the lipoprotein, promoting the detachment of ApoA-I from the HDL surface [15]. This fact could be associated with an impaired HDL function. Finally, our data also agree with previous reports of low levels of large HDLs in high CVR states [33].

Regarding LDL atherogenicity properties, LDL particles with smaller estimated size and more prone to become oxidized were associated with greater CVR scores. This concurs with previous evidence: small and oxidized LDL particles have been related to a greater coronary risk [3,4]. A lower LDL resistance against oxidation (present in coronary disease patients [19]) could facilitate LDL oxidation. Otherwise, ApoB levels, but not LDL-C concentrations, appeared to be significantly increased in high CVR states. This fact agrees with the hypothesis that alternative measurements of LDL quantity in circulation (such as ApoB levels or LDL particle number) could be more accurate and reflect better the CVR derived from these atherogenic lipoproteins [34].

Diabetes was strongly associated with dysfunctional lipoprotein characteristics in our cohort: it was associated with oxidized, small, triglyceride-rich LDL particles and with impaired cholesterol efflux capacity (although this association was lost when adjusting for HDL-C levels). Diabetes is strongly related to a suboptimal lipid profile [35] and a pro-oxidant, pro-inflammatory status that could contribute to promoting HDL dysfunctionality [36] and LDL atherogenicity [37]. The fact that there was 5% fewer dyslipidemic patients and 9.3% more individuals treated with statins in the group of diabetic individuals could contribute to explaining their lower cholesterol levels.

Once adjusted for the effect of HDL-C concentrations, being dyslipidemic was independently associated with greater CETP activity, lower HDL capacity to esterify cholesterol, and smaller HDL size, in agreement with previous work [38]. Dyslipidemia was also independently linked to LDL particles richer in triglycerides. Some authors consider this fact may be linked to a subtype of triglyceride-rich remnant lipoproteins, markedly pro-atherogenic [39].

Other classical CVRFs were shown to impair lipoprotein characteristics in our dataset. On the one hand, increased BMI values were independently associated with lower HDL-C levels, lower HDL ability to esterify cholesterol, and triglyceride-rich, small HDL particles, as well as with low estimated LDL size. Some of these lipoprotein characteristics had already been associated with excess body weight [40]. In addition, hypertriglyceridemic states in overweight or obesity could facilitate the accumulation of triglycerides in HDL particles [40], possibly leading to the formation of more dysfunctional lipoproteins [15]. On the other hand, although the associations were non-significant, our results also suggest hypertension could be related to a lower cholesterol efflux capacity and to greater LDL oxidation, potential mechanisms to be addressed in future trials.

Men are known to be more strongly affected by cardiovascular diseases than women [41], hence a potentially deleterious effect of male sex on lipoprotein traits could be expected. In our data, being male was independently associated with low HDL-C levels and, once the confounding effect of HDL-C concentrations was considered, it was also linked to greater HDL oxidation and a reduced HDL capacity to promote the endothelial release of nitric oxide, pointing to two potential novel contributors for the increased CVR in men that should be checked in further studied. In addition, male sex was linked to high concentrations of oxidized, small, cytotoxic LDL lipoproteins, but the significant of these associations was blunted when adjusting for LDL-C levels. These data agree with previous works reporting increased levels of small [42] and oxidized [43] LDL particles in men.

Aging has been traditionally associated with lower cholesterol levels, particularly in LDL, in parallel with a time-dependent increase in CVR [41,44]. Our data suggest that despite this cholesterol decrease, greater age is independently associated with a highly atherogenic LDL profile (with oxidized, small, triglyceride-rich, cytotoxic LDL particles). The possible conversion of LDL into pro-atherogenic particles could explain why CVR keeps increasing throughout life. The main strength of the present study is that it has comprehensively assessed the associations of HDL functionality and LDL atherogenicity characteristics with HDL-C and LDL-C levels, and the main factors modulating CVR. Moreover, all the relationships described in our regression models have been adjusted for the effect of the rest of CVRFs and modulators. However, there are also limitations. First, its design was cross-sectional, it did not allow us to infer causality and we could only establish associations between lipoprotein characteristics and CVRFs and modulators that should be addressed in future prospective studies. Second, our study subjects were older and at high CVR, therefore results cannot be extrapolated to the general population. To partially correct this limitation, we considered these factors as covariates in the linear regression analyses. Third, we could not perform the association analyses between CVR scores and HDL- and LDL-related characteristics in individuals aged ≥75 since the Framingham-REGICOR equation only allows the calculation of CVR scores in subjects 35 to 74 years old. Fourth, due to availability and technical issues, we were unable to analyze the HDL ability to esterify cholesterol and CETP and PON1 activities, HDL size, and HDL vasodilatory capacity in 67, 37, and 60 volunteers. Finally, we could not detect powerful associations between hypertension or smoking and lipoprotein properties since only a small proportion of our volunteers was non-hypertensive (17.6–21.3%) or a smoker (12.5–13.8%).

## Conclusions

High CVR scores were associated with low cholesterol efflux capacity values, high HDL oxidation, triglyceride-rich HDL cores, small HDL size, small estimated LDL size, and low LDL resistance against oxidation. Among high CVR subjects, being dyslipidemic and male were preferentially associated with a dysfunctional HDL profile, while being diabetic and older was specially related to pro-atherogenic LDL particles. To date, this is the first study to comprehensively analyze the independent associations between CVR and HDL- and LDL-related variables in humans. Our data reflect the pertinence of assessing HDL function and LDL atherogenicity in clinical studies, since much more information can be provided by lipoproteins beyond HDL-C and LDL-C levels.

## Supporting information

S1 Table Independent associations between CVRFs and HDL- and LDL-related variables. (DOCX)

S2 Table Independent associations between male sex and increasing 1 year of age and HDL- and LDL-related variables. (DOCX)
