# Supplementary material for: Role of HDL function and LDL atherogenicity on cardiovascular risk: A comprehensive examination
Source: PLoS One. 2019 Jun 27;14(6):e0218533. doi: 10.1371/journal.pone.0218533 (PMC6597156; doi:10.1371/journal.pone.0218533)
Supplement: S1 Table — (DOCX) [file pone.0218533.s001.docx]

**S1 Table. Independent associations between CVRFs and HDL- and LDL-related variables.**

|  | **Type-II diabetes** | | | **Dyslipidemia** | | | **Increases in 1 kg/m^2^ of BMI** | | |
| --- | --- | --- | --- | --- | --- | --- | --- | --- | --- |
|  | Model 1 | Model 2 | Model 3 | Model 1 | Model 2 | Model 3 | Model 1 | Model 2 | Model 3 |
| HDL-C levels (mg/dL) | -6.52***  [-9.05;-3.99] | -4.93***  [-7.50;-2.36] | - | 6.05***  [2.95;9.14] | 3.37*  [0.41;6.33] | - | -0.48**  [-0.82;-0.14] | -0.53**  [-0.84;-0.21] | - |
| ApoA-I levels (mg/dL) | -8.08**  [-13.6;-2.61] | -6.90*  [-12.3;-1.49] | - | 15.6***  [9.43;21.9] | 9.10**  [3.15;15.1] | - | -0.63  [-1.35;0.090] | -0.62  [-1.26;0.026] | - |
| Cholesterol efflux capacity  (normalized ratio) | -0.041**  -0.065;-0.017] | -0.027*  [-0.052;-0.003] | -0.010  [-0.033;0.013] | 0.059***  [0.031;0.088] | 0.042**  [0.014;0.070] | 0.022  [-0.004;0.049] | -0.001  [-0.004;0.002] | -0.001  [-0.004;0.002] | 7·10^-4^  [-0.002;0.003] |
| HDL cholesterol esterification  Index (unitless ratio) | -0.002  [-0.49;0.49] | -0.18  [-0.72;0.35] | -0.14  [-0.68;0.41] | -0.73*  [-1.31;-0.15] | -0.58  [-1.17;0.001] | -0.67*  [-1.27;-0.066] | -0.084**  [-0.15;-0.022] | -0.066*  [-0.13;-0.004] | -0.068*  [-0.13;-0.005] |
| CETP activity  (normalized ratio) | -0.065*  [-0.12;-0.008] | -0.003  [-0.067;0.062] | -0.004  [-0.070;0.062] | 0.085*  [0.016;0.16] | 0.069  [-0.003;0.14] | 0.080*  [0.006;0.15] | 0.001  [-0.006;0.008] | -7·10^-4^  [-0.008;0.007] | -3·10^-4^  [-0.008;0.007] |
| PON1 arylesterase activity  (normalized ratio) | -0.051  [-0.16;0.052] | -0.034  [-0.15;0.082] | -0.026  [-0.14;0.093] | 0.074  [-0.050;0.20] | 0.057  [-0.071;0.19] | 0.030  [-0.10;0.16] | -0.007  [-0.020;0.006] | -0.009  [-0.022;0.005] | -0.007  [-0.021;0.007] |
| HDL vasodilatory capacity  (normalized ratio) | 0.027  [-0.056;0.11] | 0.022  [-0.064;0.11] | 0.006  [-0.081;0.094] | 0.050  [-0.050;0.15] | 0.009  [-0.089;0.11] | 0.021  [-0.079;0.12] | 0.004  [-0.007;0.015] | 0.002  [-0.009;0.012] | -4·10^-4^  [-0.011;0.010] |
| HDL oxidation  (normalized ratio) | 0.46*  [0.047;0.87] | 0.086  [-0.36;0.54] | 0.095  [-0.38;0.57] | 0.12  [-0.37;0.62] | 0.14  [-0.37;0.65] | 0.15  [-0.39;0.69] | 0.017  [-0.037;0.072] | 0.018  [-0.037;0.073] | 0.019  [-0.038;0.076] |
| Triglycerides in HDL core  (unitless ratio) | 0.032  [-0.010;0.074] | 0.036  [-0.010;0.083] | 0.009  [-0.033;0.052] | 4·10^-4^  [-0.049;0.050] | -0.008  [-0.061;0.045] | 0.022  [-0.026;0.071] | 0.007*  [0.001;0.012] | 0.007*  [0.002;0.013] | 0.004  [-6·10^-4^;0.010] |
| HDL_2_/HDL_3_  (unitless ratio) | 0.018  [-0.036;0.073] | -0.013  [-0.071;0.044] | 0.020  [-0.035;0.075] | -0.022  [-0.088;0.043] | -0.049  [-0.11;0.016] | -0.073*  [-0.14;-0.011] | -0.014***  [-0.021;-0.007] | -0.012***  [-0.019;-0.006] | -0.008*  [-0.015;-0.002] |
| LDL-C levels (mg/dL) | -15.4***  [-23.9;-6.87] | -13.8**  [-22.1;-5.47] | - | 29.9***  [20.1;39.6] | 28.1***  [18.5;37.7] | - | -0.14  [-1.26;0.97] | -0.70  [-1.72;0.32] | - |
| ApoB (mg/dL) | -7.27*  [-13.9;-0.66] | -5.93  [-12.8;0.99] | - | 19.8***  [12.3;27.3] | 18.0***  [10.2;25.8] | - | 0.49  [-0.38;1.36] | 0.46  [-0.39;1.30] | - |
| LDL size (LDL-C/ApoB)  (unitless ratio) | -0.088***  [-0.13;-0.050] | -0.072***  [-0.11;-0.030] | -0.046*  [-0.084;-0.007] | 0.051*  [0.002;0.10] | 0.032  [-0.015;0.079] | -0.017  [-0.062;0.029] | -0.005  [-0.01;7·10^-4^] | -0.007**  [-0.012;-0.002] | -0.007**  [-0.012;-0.002] |
| LDL oxidation  (normalized ratio) | 0.26***  [0.13;0.39] | 0.21**  [0.080;0.34] | 0.12* [0.016;0.23] | -0.11  [-0.27;0.050] | -0.090  [-0.24;0.057] | 0.084  [-0.050;0.22] | 0.006  [-0.012;0.023] | 0.006  [-0.010;0.021] | -0.004  [-0.017;0.009] |
| LDL lag time  (normalized ratio) | -0.070**  [-0.12;-0.019] | -0.061  [-0.12;-0.007] | -0.047  [-0.10;0.010] | 0.019  [-0.042;0.080] | 0.026  [-0.038;0.089] | 3·10^-4^  [-0.072;0.072] | 0.001  [-0.005;0.008] | 0.002  [-0.005;0.009] | 0.003  [-0.004;0.010] |
| Triglycerides/cholesterol  in LDLs (unitless ratio) | 0.018*  [0.002;0.034] | 0.018*  [0.002;0.035] | 0.012  [-0.004;0.028] | 0.010  [-0.009;0.029] | 0.001  [-0.018;0.020] | 0.021*  [0.002;0.040] | 0.002  [-4·10^-4^;0.004] | 0.001  [-8·10^-4^;0.003] | 6·10^-4^  [-0.001;0.002] |
| LDL cytotoxicity in macrophages  (normalized ratio) | 0.65*  [0.084;1.21] | 0.49  [-0.10;1.08] | 0.36  [-0.24;0.96] | -0.16  [-0.84;0.53] | -0.096  [-0.77;0.57] | 0.38  [-0.35;1.10] | -0.032  [-0.10;0.040] | -0.032  [-0.10;0.039] | -0.045  [-0.12;0.026] |

CVRFs indicates cardiovascular risk factors; ApoA-I, apolipoprotein A-I; ApoB, apolipoprotein B; BMI, body mass index; CETP, cholesteryl ester transfer protein; HDL-C, HDL cholesterol; LDL-C, LDL cholesterol; PON1, paraoxonase-1. Data are beta coefficients [95% CI] obtained in multivariate linear regression analysis, non-adjusted (Model 1), adjusted for the rest of cardiovascular risk factors, study site, adherence to a Mediterranean Diet, and levels of physical activity (Model 2), and for all the previous factors plus HDL-C or LDL-C levels (in HDL- or LDL-related variables, respectively) (Model 3). *: *P*<0.05; **: *P*<0.01; ***: *P*<0.001.
