# Supplementary material for: Role of HDL function and LDL atherogenicity on cardiovascular risk: A comprehensive examination
Source: PLoS One. 2019 Jun 27;14(6):e0218533. doi: 10.1371/journal.pone.0218533 (PMC6597156; doi:10.1371/journal.pone.0218533)
Supplement: S2 Table — (DOCX) [file pone.0218533.s002.docx]

**S2 Table. Independent associations between male sex and increasing 1 year of age and HDL- and LDL-related variables.**

|  | **Male sex** | | | **Increases in 1 year of age** | | |
| --- | --- | --- | --- | --- | --- | --- |
|  | Model 1 | Model 2 | Model 3 | Model 1 | Model 2 | Model 3 |
| HDL-C levels (mg/dL) | -9.24***  [-11.7;-6.83] | -7.80***  [-10.3;-5.27] | - | 0.19  [-0.015;0.39] | 0.084  [-0.11;0.27] | - |
| ApoA-I levels (mg/dL) | -19.5***  [-24.4;-14.5] | -15.5***  [-20.7;-10.2] | - | 0.37  [-0.036;0.78] | 0.072  [-0.30;0.45] | - |
| Cholesterol efflux capacity  (normalized ratio) | -0.056***  [-0.080;-0.033] | -0.038**  [-0.062;-0.014] | -0.010  [-0.034;0.013] | 0.001  [-7·10^-4^;0.003] | 8·10^-4^  [-0.001;0.003] | 4·10^-4^  [-0.001;0.002] |
| HDL cholesterol esterification  Index (unitless ratio) | 0.59*  [0.10;1.08] | 0.36  [-0.15;0.87] | 0.39  [-0.17;0.94] | 0.014  [-0.024;0.052] | 0.028  [-0.013;0.068] | 0.024  [-0.017;0.064] |
| CETP activity  (normalized ratio) | -0.081**  [-0.14;-0.023] | -0.068*  [-0.13;-0.006] | -0.066  [-0.13;6·10^-4^] | -0.004  [-0.008;7·10^-4^] | -0.004  [-0.009;0.001] | -0.003  [-0.008;0.002] |
| PON1 arylesterase activity  (normalized ratio) | -0.058  [-0.16;0.045] | -0.061  [-0.17;0.051] | -0.032  [-0.15;0.088] | -0.009*  [-0.016;-7·10^-4^] | -0.008  [-0.017;6·10^-4^] | -0.009  [-0.017;2·10^-4^] |
| HDL vasodilatory capacity  (normalized ratio) | -0.014  [-0.096;0.069] | -0.038  [-0.12;0.048] | -0.094*  [-0.19;-0.002] | 0.002  [-0.004;0.008] | 0.004  [-0.003;0.010] | 0.004  [-0.002;0.011] |
| HDL oxidation  (normalized ratio) | 0.48*  [0.063;0.89] | 0.55*  [0.098;0.99] | 0.56*  [0.071;1.05] | 0.002  [-0.030;0.034] | -0.001  [-0.034;0.032] | -0.001  [-0.035;0.033] |
| Triglycerides in HDL core  (unitless ratio) | 0.018  [-0.024;0.060] | 0.026  [-0.020;0.071] | -0.039  [-0.082;0.005] | 0.002  [-0.001;0.005] | 0.002  [-0.001;0.006] | 0.003*  [3·10^-4^;0.006] |
| HDL_2_/HDL_3_  (unitless ratio) | -0.068*  [-0.12;-0.015] | -0.083**  [-0.14;-0.027] | -0.028  [-0.084;0.029] | 0.007*** [0.003;0.012] | 0.004*  [1·10^-4^;0.008] | 0.004  [-4·10^-5^;0.008] |
| LDL-C levels (mg/dL) | -14.8***  [-23.3;-6.23] | -6.44  [-14.7;1.79] | - | -0.87**  [-1.52;-0.22] | -1.13***  [-1.77;-0.49] | - |
| ApoB (mg/dL) | -6.16  [-12.8;0.52] | -2.03  [-8.73;4.66] | - | -0.51*  [-0.99;-0.041] | -0.64*  [-1.14;-0.14] | - |
| LDL size (LDL-C/ApoB)  (unitless ratio) | -0.062**  [-0.10;-0.022] | -0.046*  [-0.086;-0.006] | -0.033  [-0.070;0.004] | -0.002  [-0.005;0.001] | -0.002  [-0.005;0.001] | -3·10^-4^  [-0.003;0.003] |
| LDL oxidation  (normalized ratio) | 0.085  [-0.051;0.22] | 0.13*  [0.005;0.26] | 0.028  [-0.079;0.13] | 0.013*  [0.003;0.024] | 0.013**  [0.003;0.023] | 0.003  [-0.005;0.012] |
| LDL lag time  (normalized ratio) | -0.017  [-0.068;0.035] | -0.001  [-0.056;0.053] | 0.006  [-0.050;0.063] | 0.002  [-0.001;0.006] | 0.003  [-0.002;0.007] | 0.003  [-0.001;0.008] |
| Triglycerides/cholesterol  in LDLs (unitless ratio) | -0.026**  [-0.042;-0.010] | -0.022**  [-0.038;-0.006] | -0.027*  [-0.042;-0.012] | 0.002*  [3·10^-4^;0.003] | 0.001*  [9·10^-5^;0.003] | 6·10^-4^  [-6·10^-4^;0.002] |
| LDL cytotoxicity in macrophages  (normalized ratio) | 0.41  [-0.16;0.98] | 0.61*  [0.033;1.19] | 0.38  [-0.20;0.96] | 0.043  [-3·10^-4^;0.086] | 0.050*  [0.005;0.095] | 0.030  [-0.016;0.075] |

ApoA-I indicates apolipoprotein A-I; ApoB, apolipoprotein B; BMI, body mass index; CETP, cholesteryl ester transfer protein; HDL-C, HDL cholesterol; LDL-C, LDL cholesterol; PON1, paraoxonase-1.

Data are beta coefficients [95% CI] obtained in multivariate linear regression analysis, non-adjusted (Model 1), adjusted for the rest of cardiovascular risk factors, study site, adherence to a Mediterranean Diet, and levels of physical activity (Model 2), and for all the previous factors plus HDL-C or LDL-C levels (in HDL- or LDL-related variables, respectively) (Model 3). *: *P*<0.05; **: *P*<0.01; ***: *P*<0.001.
